# Assessment of nurses’ cardiopulmonary resuscitation knowledge and skills within three district hospitals in Botswana

**DOI:** 10.4102/phcfm.v10i1.1633

**Published:** 2018-04-12

**Authors:** Lakshmi Rajeswaran, Megan Cox, Stoffel Moeng, Billy M. Tsima

**Affiliations:** 1 School of Nursing, University of Botswana, Botswana; 2 Department of Emergency Medicine, University of Botswana, Botswana; 3 Department of Statistics, University of Botswana, Botswana; 4 Department of Family Medicine & Public Health, University of Botswana, Botswana

## Abstract

**Background:**

Nurses are usually the first to identify the need for and initiate cardiopulmonary resuscitation (CPR) on patients with cardiopulmonary arrest in the hospital setting. Cardiopulmonary resuscitation has been shown to reduce in-hospital deaths when received from adequately trained health care professionals.

**Aim:**

We aimed to investigate nurses’ retention of CPR knowledge and skills at district hospitals in Botswana.

**Methods:**

A quantitative, quasi-experimental study was conducted at three hospitals in Botswana. A pre-test, intervention, post-test, and a re-test after 6 months were utilised to determine the retention of CPR knowledge and skills. Non-probability, convenience sampling technique was used to select 154 nurses.

The sequences of the test were consistent with the American Heart Association’s 2010 basic life support (BLS) guidelines for health care providers. Data were analysed to compare performance over time.

**Results:**

This study showed markedly deficient CPR knowledge and skills among registered nurses in the three district hospitals. The pre-test knowledge average score (48%) indicated that the nurses did not know the majority of the BLS steps. Only 85 nurses participated in the re-evaluation test at 6 months. While a 26.4% increase was observed in the immediate post-test score compared with the pre-test, the performance of the available participants dropped by 14.5% in the re-test 6 months after the post-test.

**Conclusion:**

Poor CPR knowledge and skills among registered nurses may impede the survival and management of cardiac arrest victims. Employers and nursing professional bodies in Botswana should encourage and monitor regular CPR refresher courses.

## Introduction

Cardiopulmonary resuscitation (CPR) is a well-recognised medical procedure in which chest compressions and artificial ventilation are provided to maintain adequate blood flow to the brain and other vital organs.^1^ Cardiopulmonary resuscitation has been shown to reduce in-hospital cardiac death and related fatalities when patients are managed by adequately trained health care professionals.^2^ The American Heart Association (AHA) is the leading authority on resuscitation science. Its approved training courses are taught across the globe. In an effort to practise evidence-based medicine, AHA updates are released every 5 years. The 2015 AHA update for CPR and emergency cardiovascular care (ECC) focuses on topics involving significant new developments in resuscitation science or ongoing controversies, and serves as an update to the 2010 AHA Guidelines for CPR and ECC rather than as a complete revision of the guidelines.^3^

Nurses are often the first health care professionals to identify a patient with cardiopulmonary arrest in the hospital setting and therefore should possess adequate competency to provide effective resuscitation. Cardiopulmonary resuscitation has been practised for over 50 years and many studies have shown that knowledge and skills decline within 6 months after initial training in CPR ^4,5^ and performance improves when all nurses are certified and practising the relevant life support training courses.^6^

The quality of CPR performed by rescuers depends on learners integrating, retaining and applying the cognitive, behavioural and psychomotor skills required to successfully perform resuscitation.^7^

This study aimed to identify the baseline knowledge, skills and knowledge retention for CPR among registered nurses in three selected district hospitals in Botswana, an upper middle-income country in southern Africa.

There is little data from Africa regarding nurses’ knowledge and skills in CPR, especially from district hospitals. Previous studies in Botswana measured the retention of CPR knowledge and skills among health care workers following a training course in a single district and two referral hospitals, respectively.^8,9^ Both studies revealed that health care providers retained the knowledge and skills for 3 months after training, but the retention did not last for 6 months for adult CPR skills. We aimed to assess registered nurses’ knowledge and CPR skills in three different district hospitals in Botswana recently engaged in an external health services accreditation process.

## Research methods and design

The study employed a quantitative design with the knowledge and skills response scored by the research team members. The study used a pre-test, intervention, a post-test and a re-test after 6 months design to determine the retention of CPR knowledge and skills among registered nurses. Same participants were assessed at baseline and at subsequent visits thus acting as their own study controls.

This study was conducted in three district hospitals in Botswana undergoing accreditation processes which require basic life support (BLS) certification as one of the skills monitored. These district hospitals serve populations of approximately 50 000 to 70 000 and admit all general medical, surgical, paediatric and obstetrics patients.

Data were analysed using the Statistical Package of Social Sciences (SPSS) version 25 program. The Wilcoxon Matched-Pairs Signed Rank Non-Parametric Test examined changes that occurred between the pre-test and post-test measures, and a *p*-value < 0.05 was considered to be statistically significant.

Non-probability, convenience sampling technique was used to select 154 nurses from Accident and Emergency wards; Intensive Care Units; Male and Female Medical wards; and Surgical, Orthopaedic, Obstetrics and Gynaecology wards.

We included consenting nurses registered with the Nursing and Midwifery Council of Botswana (NMCB) currently practising in the selected hospitals. The NMCB registers nurses who have completed 3 years of general nursing training and nurses who have completed post-basic midwifery training programme for 18 months as registered nurses and midwives, respectively.

An initial survey document collected demographic data such as age, gender, professional status, academic qualification, work experience and prior CPR training. Knowledge of CPR and BLS was then obtained in a 20-item multiple choice questionnaire (MCQ). The questions were based on theory from the AHA 2010 BLS guidelines and were pre-tested on 15 nurses who did not participate in the main study. A pass mark of 85% for the questionnaire was adopted in our study, as this is consistent with the official AHA guidelines.

Cardiopulmonary resuscitation skills were assessed individually by an investigator at a separate time and place from the questionnaire administration. Investigators who were current AHA-certified BLS instructors assessed all nurses’ performances. During pre-test (before training), knowledge was assessed by using the 20-item MCQ while CPR skills were assessed in individual nurses independently using an adult training manikin. The sequence of tests included the theoretical test followed by skills testing consistent with the AHA 2010 BLS guidelines for health care providers. Three investigators assessed nurses’ CPR skills using the critical performance criteria outlined in the AHA 2010 BLS for health care providers. Assessors observed five cycles of CPR using the same tool. Theoretical aspects covered the core concepts of CPR included in the AHA 2010 CPR guidelines and was delivered through a 30-min didactic session. The instructors demonstrated CPR skills for 60 min maintaining a ratio of three nurses, one manikin and one instructor during the training sessions. Twelve nurses attended each training session.

Participants’ knowledge and skills were assessed immediately after the training and a re-test was conducted after 6 months to re-evaluate the retention of their CPR knowledge and skills. The same MCQ and skills assessment tool were used in all three settings by the same instructors during pre-, post- and re-test assessment to maintain consistency and uniformity in assessment.

The Laerdal™ ‘little Anne’ CPR training manikin and a training Automated External Defibrillator (AED) were used to assess the registered nurses’ CPR skills. The CPR skills were assessed following 16 steps and were graded as correct and incorrect. Two BLS instructors assessed each participant’s skills independently and the scores were consolidated at the end. Only adult cardiac arrest protocols were used in our study.

### Ethical considerations

Permission to conduct the study was granted by the University of Botswana Institutional Review Board (UBR/RES 3/2), the Botswana Ministry of Health (PPME-13/18/1 VIII[189]) and all the three district hospitals’ research committees. Participation was voluntary and informed consent was obtained from each participant. All participants were assigned a code number and the same numbers were maintained during the pre-, post- and re-testing the knowledge and skills. Data were kept in a safe place and only the research team had access to the data. Furthermore, only the questionnaire number and the anonymous codes were captured and separated from the main data during the analysis.

## Results

Of our study population of 154 nurses, the majority (70%) were female. Nearly 60% were aged between 20 and 30 years and 18% were aged over 40 years. Over 84% of the nurses were trained to diploma level (nursing or midwifery or both); graduate nurses represented 16%; and only one nurse had obtained a master’s qualification.

The duration of work experience was divided between 49.3% (working from between 1 and 6 years) and 50.6% (working for more than 7 years). Areas of work were distributed through all departments of the district hospitals apart from operating theatres. Previous in-hospital experience of CPR training was also evenly split with 52% reporting no prior formal CPR training and 48% reporting some previous CPR or BLS training.

In the pre-test CPR knowledge MCQ, the majority (51.3%) of participants scored less than 50%, and 87% of participants scored less than 70%. Only 4% achieved the 85% pass rate recommended by the AHA and the pre-test mean score was 48%. Particularly poorly answered questions were on the initial steps in BLS, management of sudden collapse and the five links in the chain of survival.

Scores from the post-test immediately after the training revealed a considerable improvement with a mean score of 74.2%, significantly higher than the mean of 48.2% at pre-test (*p* < 0.001) as shown in Table 1.

**TABLE 1:** Descriptive statistics of total score (%) of pre-test and post-test basic life support multiple choice questionnaire.

Variables	*N*	Minimum	Median	Maximum	Mean	Standard deviation
Pre-test	154	10.00	45.00	95.00	48.15	17.02
Post- test	154	30.00	75.00	100.00	74.16	13.24

Male participants scored slightly better than their female counterparts (52.9% vs. 46.1%) in the pre-test. However, there was no difference between the genders in the post-test (74.2% vs. 74.0%). In addition, males performed better than females (63.2% vs. 59.8%) in the re-test after 6 months.

Only 85 nurses (55%) from the original 154 were available for the 6-month post-test. The available participants’ CPR questionnaire performance dropped by 14.5% in the re-test in 6 months’ time after initial training.

The knowledge level of nurses in all the three hospitals was almost the same in the pre-, post- and re-tests. While the majority scored between 45% and 51% in the pre-test, there was a considerable improvement in the scores in all three centres after the training session (72.9%–76.4%). Performance dropped again in all groups after 6 months in the re-test to between 60% and 62.4%.

The overall initial mean score for the CPR skills tests was lower than those of the CPR knowledge tests, but the change after training was greater. The mean score of the nurses’ CPR skills before training was 18.3% but post-test mean score increased to 79.5% as shown in Table 2. After 6 months, the score decreased to 51.5% in those available to be tested.

**TABLE 2:** Comparative statistics of scores on cardiopulmonary resuscitation skills between pre-test and post-test.

Variables	*N*	Minimum	Median	Maximum	Mean	Standard deviation
Pre-test	154	0.00	6.25	100.00	18.30	28.97
Post-test	154	0.00	93.75	100.00	79.50	29.89
Re-test	85	0.00	50.00	100.00	54.00	28.68

**FIGURE 1:**
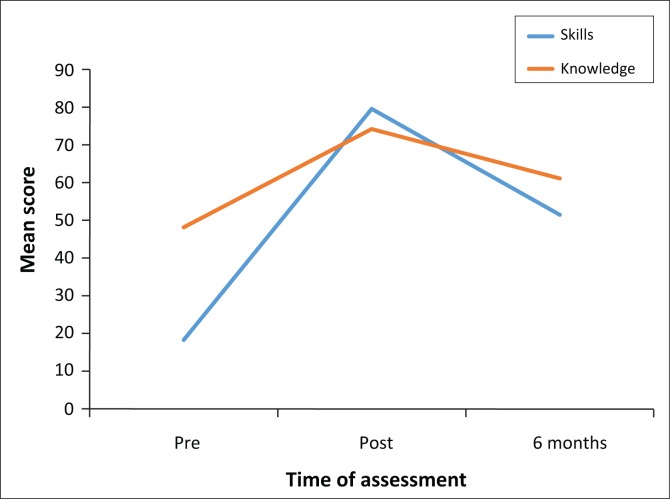
Performance of participants in all hospitals in the pre-, post- and re-tests both on cardiopulmonary resuscitation knowledge and skills.

Overall performance in all the testing, both written and practical, is shown in Figure 1.

The difference in performance, total score between pre-test and post-test between the age groups, academic qualifications and years of experience were not statistically significant. The highest improvement realised (28.5%) was observed among the participants with 5–6 years of experience and the least improvement (22.7%) was among the participants who have more than 11 years of experience.

## Discussion

### Pre-test knowledge of cardiopulmonary resuscitation

This study showed markedly deficient CPR knowledge and skills among registered nurses in the three district hospitals in a resource-limited setting. The pre-test knowledge score (48.2%) of 154 registered nurses indicated that they did not know the correct compression ventilation ratio, compression rate, updated AHA guidelines, BLS steps, chain of survival and the indication for using an automated external defibrillator (AED) and providing ventilation using the bag-valve mask. These are the critical aspects of CPR and incorrect steps in initiating CPR reduce the chances of survival after a cardiac arrest.^10,11^ A previous study conducted in the two referral hospitals in Botswana also identified a huge gap in nurses’ knowledge and skills in CPR.^9^ This previous and current study conducted in Botswana indicate that regardless of the setting of the hospitals, CPR knowledge and skills remain poor among nurses working in different areas. These findings are not unique to nurses working in Botswana. Other authors concur that the knowledge level on critical skills deteriorates gradually within 6 months.^12,13^ Nurses’ knowledge levels have also been noted to be influenced by their motivation, attitude and willingness to learn BLS or participate in drills.^14^

Cardiopulmonary resuscitation skills of the registered nurses were poor during pre-test and no one could pass the essential skills such as checking the scene safety, checking the pulse, activating the emergency response system, giving high-quality CPR, ensuring adequate compression rate, operating the AED, using the bag-valve mask and administering defibrillation to the cardiac arrest victims. Studies conducted in Turkey^12^ and Saudi Arabia^15^ also revealed that nurses were not able to perform early defibrillation in a timely fashion and also perform bag-valve mask ventilation.^16^ Lack of skills among nurses in providing ventilation during cardiac arrest by bag-valve mask could result in hyperventilating the cardiac arrest victim. Ventilating patients at an appropriate rate while CPR is ongoing is important and should be emphasised during all resuscitation training.^17^

The AED should be considered for the hospital setting as a way to facilitate early defibrillation especially in developing countries where most of the health care providers have no rhythm recognition skills or where the defibrillators are used infrequently.^18^ The United Kingdom Resuscitation Council recommends that nurses and student nurses should be trained to use the AED to facilitate early defibrillation and the AED should be available in all non-critical wards.^19^ Competence in defibrillation (both AED and manual defibrillation) calls for periodical training requiring repeated practice.^20^ Teaching of the necessary skills, such as assessing the unconscious victim, and beginning early CPR until the arrival of the emergency response system may improve the survival rate and reduce the hospital mortality rate.^21^ In the present study, the fact that the nurses had deficient knowledge and skills in using AED and bag-valve mask, and in compressing the chest adequately, might be because of inadequate training in the district hospitals. The knowledge gaps may exist because of lack of availability of AED in these hospitals. Because of resource limitations at these hospitals, AED availability is inconsistent across the hospital. Future studies should evaluate the impact of availability of AED on nurses’ CPR knowledge and skill.

### Improvement after initial training

The present study confirms that nurses are able to demonstrate significant improvement in their knowledge rates after training when one compares their initial results in the pre-test with the results obtained in the post-test. A study conducted in Iran confirmed that regular training and workshops can be useful and effective to help retain nurses’ knowledge and skills.^22^ Other CPR researchers point out that when a lengthy interval occurs between the periodic CPR, refresher-training courses should be provided to all the practising nurses as a part of their continuous professional development.^23^

### Retention at 6 months (re-testing)

Research has shown that health care workers’ knowledge and CPR skills decrease in 3 months’ time after training.^12,15^ In this study, re-assessment at 6 months showed a knowledge level decline to 14.5%, indicating that nurses did not retain the information provided during their training. The skills level dropped to 26.1% also indicating that nurses did not retain the necessary skills applied during CPR. The theoretical questions on assessing the pulse for the infant, witnessed collapse for the child, two rescuers CPR and the chain of survival were still responded to poorly at re-testing. This underscores the need for nurses to undergo regular training so as to ensure skill retention. Such training could include regular hospital-based CPR drills to promote skill retention. Our findings indicate that participants in the age group of over 40 years performed worse than the younger age groups. This may be due to the fact that the older age group had inadequate exposure earlier in their career owing to a lack of training resources and CPR training opportunities. This finding is similar to the findings from elsewhere that older health care providers obtained lower scores than younger health care providers during the course of being evaluated for their advance life support knowledge.^24^

In Botswana, CPR is still a developing science and recently gaining momentum as most of the hospitals in the country are undergoing accreditation processes and increasingly serving more patients with non-communicable diseases. The current study showed that male participants’ performance was better in pre-test and re-test after 6 months. An observational study conducted in a simulated environment in Germany also showed that the males performed deeper compressions than the female participants.^24^ In contrast, in a study conducted in Kenya, age, gender and work experience did not have any significant impact in CPR performance among nurses.^25^ However, there are no large, well-conducted studies available in the literature describing the effect of gender in real-life resuscitation situations and thus future studies should evaluate gender differences in CPR performance.

Nurses with a post-basic qualification performed better in pre-test and post-test. However, retention level was not satisfactory after 6 months. Participants with more than 7 years’ experience showed decreased level of knowledge after 6 months compared with those with less experience. It has previously been observed that while work experience may increase the confidence level of individual nurses, there is no correlation between years of work experience and competencies in the performance of CPR.^26^

In our study, participants who underwent in-service education and BLS training performed better than nurses who never had any exposure to BLS training programmes. This is also supported by recent studies conducted in India^26^ and in Brazil,^27^ where nurses with BLS training performed better than the nurses who never had any training.

In our study, area of assignment did not show any difference in the performance of the CPR among nurses. Other authors have conversely reported that nurses working in high-risk areas such as Intensive Care Unit (ICU) and nurses who work continuously in close contact with patients are more motivated to maintain their competence in CPR than other health care professionals.^22^ Unlike these authors, nurses working in ICU were not included in our study owing to a lack of ICU in district hospitals in Botswana.

### Limitations

Our study is not without limitations. Firstly, the study examined only three district hospitals out of eight in Botswana. Therefore, the results cannot be generalised to the registered nurses working in other district hospitals. The study was also conducted in a simulated environment rather than observing the nurses performing CPR in a real-life situation.

Furthermore, the teaching sessions, both theory and practice, were conducted within a span of one and a half hours unlike the usual day-long AHA BLS course. This may impact on the validity of the training. Thus, caution should be exercised when interpreting our results as our training programme has significant differences with the AHA standard course. Our training was designed to be shorter to minimise disruption in service as most of the nurses participating in the study were expected to return to the work area as soon as possible.

There was a high dropout rate of participants between the initial pre-test, post-test phase and the re-test 6 months later. As many as 69 of the registered nurses were either transferred to other facilities or had left to work in the private sector.

## Conclusion

The findings of the study indicate that it is imperative for registered nurses to receive regular, periodic, in-service CPR courses as well as engage in regular CPR drills to update their knowledge and skills and to be aware of changes made in the latest guidelines in CPR science.

Optimally, the frequency of the training should be once every 6 months to avoid deterioration in CPR knowledge and skills.

The poor CPR knowledge and skills among registered nurses may impede the initiation of CPR for cardiac arrest victims. Irrespective of their experience, areas of work and educational level, nurses have a professional responsibility to maintain competence in CPR by attending regular refresher courses.
